# Efficacy of Contact Needle Therapy for Chemotherapy-Induced Peripheral Neuropathy

**DOI:** 10.1155/2013/928129

**Published:** 2013-05-20

**Authors:** Keiko Ogawa, Masao Ogawa, Koji Nishijima, Masaki Tsuda, Genichi Nishimura

**Affiliations:** ^1^Department of Otorhinolaryngology and Head and Neck Surgery, Clinic of Japanese Oriental (Kampo) Medicine, Kanazawa University Hospital, 13-1 Takaramachi, Kanazawa-City, Ishikawa 920-8641, Japan; ^2^Department of Anesthesiology, Kanazawa Medical University Hospital, 1-1, Daigaku, Uchinada, Kahoku-gun, Ishikawa 920-0265, Japan; ^3^Department of Surgery, Japanese Red Cross Kanazawa Hospital, Mima 2-251, Kanazawa 921-8162, Japan; ^4^Mukeido Acupuncture and Moxibustion Office, Niku 126-7, Horikawa, Koizumi-cho, Toyama 939-8081, Japan

## Abstract

Cancer chemotherapy-induced peripheral neuropathy (CIPN) often results in discontinuation of treatment with potentially useful anticancer drugs and may deteriorate the patient's quality of life. This study investigated the effect of contact needle therapy (CNT) on CIPN caused by responsible chemotherapeutic agents as taxanes and oxaliplatin. Six patients with CIPN were treated with CNT. The severity of CIPN was evaluated using the Common Terminology Criteria for Adverse Events (CTCAE) version 4 and FACT/GOG-Ntx before and after CNT. After the treatment, all of the patients showed some improvement. Four patients showed apparent improvement in breakthrough pain. One of the cases had difficulty in walking because of CIPN in lower extremities, but after 2 times of CNT, he could walk without pain and could continue the chemotherapy. Although its putative mechanisms remain elusive, CNT has strong potential as an adjunctive therapy in CIPN. Well-designed clinical trials with adequate sample size and power are necessary to confirm the findings of this study.

## 1. Introduction

With the increasing numbers of patients with cancer and cancer survivors and the development of multidisciplinary cancer therapy, treatment that considers the quality of life (QOL) of patients together with prognostic improvement is demanded. Multidisciplinary cancer therapy consists of surgical treatment, radiotherapy, and chemotherapy. Chemotherapy often causes side effects such as myelosuppression, digestive symptoms, renal failure, or peripheral neuropathy. Cancer chemotherapy-induced peripheral neuropathy (CIPN) is one of the most serious problems in clinical practice, and it sometimes results in the discontinuation of subsequent treatment [1, 2]. CIPN is well known in taxanes, platinum analogues, vinca alkaloids, and molecular target drugs such as bortezomib [3]. Neuropathy by taxanes stems from damage to microtubules of the neuraxis, mainly developing from gloves-and-socks type sensory disturbance [4]. Platinum analogues such as cisplatin and oxaliplatin damage nerve cells directly, followed by damage to the neuraxis [5]. Moreover, with oxaliplatin, acute accumulation-related disorder is detected, and acute peripheral neuropathy is induced by a low-temperature stimulus that is known to be reinforced [6]. To prevent CIPN, calcium and magnesium infusions, glutathione, and anticonvulsants (carbamazepine) are used, but their effects are limited. Also, when CIPN appears, anticonvulsants, tricyclic antidepressants, alpha-lipoic acid, and opioids are often used [2, 7], but their effects may not be enough. Abrogation of chemotherapy is required to prevent the exacerbation of symptoms. Specific and effective curative treatments are lacking.

Acupuncture is a frequently used alternative and complementary medicine in Japan. Contact needle therapy (CNT) is one of the traditional Japanese methods of acupuncture, which was thought up by Bunkei Ono. We use disposable needle and do not insert at all but only settle the needle on the acupuncture point to perform the least but effective stimulus to unblock the meridian. The aim of CNT is to improve the patients' condition no matter what their diseases are by regulating the flow of Qi. This method has many advantages such that it is safe, painless, easy to perform, and it decreases the risk of infection.

From ancient times, it has been said that the larger the needle is and the deeper the needle is inserted, the stronger the stimulation will be. If the stimulation is too strong, the patients' condition becomes worse, especially when their constitution is weak. CNT is known as a method of weak stimulation. In this aspect, CNT is effective and appropriate for treating cancer patients. It is also expected to relieve symptoms such as the side effects of chemotherapy in several clinical reports in Japanese [8].

Recently, clinical trials of acupuncture for prevention or treatment of CIPN have been conducted [9]. Publications in English language journals on acupuncture as a symptomatic treatment for CIPN have been limited to only a few case studies, all of which report an improvement in symptoms [10–13]. But there is no report of CNT and it has never been clinically evaluated in English publications, because CNT may have been considered as placebo acupuncture.

In this study, we investigated whether CNT had beneficial effects in patients with CIPN after or during chemotherapy.

## 2. Patients and Methods

### 2.1. Preliminary Study

#### 2.1.1. Patients

Between July 2012 and January 2013, acupuncture treatment was offered to all the patients who were diagnosed to have CIPN by surgeons in Japanese Red Cross Kanazawa Hospital. Seven patients agreed to receive acupuncture treatment, CNT. We excluded 1 patient whose CIPN had been partially caused by uncontrolled diabetes mellitus from this study. Therefore, we evaluated six patients with CIPN. We checked the patients' background (age, sex, and cancer types) and contents of chemotherapy. Six patients (four men and two women) of mean age 64.3 years received the best medical care and additionally were treated with CNT for CIPN. The types of cancer were one case of breast cancer and 5 cases of colorectal cancer.

#### 2.1.2. Contact Needle Therapy (CNT)

The specific acupuncture protocols employed in this study are described later, point location were as described in standard textbooks [14], and disposable sterile silver needles of 0.16 × 24 mm were used and left in place for 30 second to 1 min without insertion. Each patient received standard 4–6 treatments in 3 months.

Acupuncture was performed in all cases by the same senior acupuncturist who had used acupuncture for 20 years. CNT was performed to the patients according to the medical diagnosis of meridian therapy. Acupuncture points used in this study are as follows.

Points for all patients: CV12, CV4, ST25, KI2, well points of extremities.

Selected points: LR8, LR14, SP3, LR13, LU9, LU1, KI7, GB25, PC7, CV17, CV6, CV4, ST36, LU1, BL20, BL13, BL18, BL23.

#### 2.1.3. Endpoints and Evaluation

The severity of CIPN was graded using the Common Terminology Criteria for Adverse Events (CTCAE) version 4.0 and also FACT/GOG-Ntx [15]. Clinical evaluation was performed before and after CNT because of the limitation of CTCAE for precise evaluation of symptoms. Patients' objective evaluations were also obtained.

## 3. Results

### 3.1. Preliminary Results in 6 Patients

Table 1 shows the patient characteristics. The mean age of the 6 patients was 64.3 years old (range: 54–73). Each patient had undergone an operation with chemotherapy. Four of 6 received concurrent chemotherapy, but all of them excluded responsible chemotherapeutic agents from regimens for 1–26 months before the start of CNT. Five of 6 are tumor-bearing status, and case 2 is receiving chemotherapy as adjuvant setting. As chemotherapeutic agents responsible for peripheral neuropathy, taxanes were used in Patient 2, and in others platinum analogue oxaliplatins were used.

Prior to CNT, the patients had grades 1-2 CIPN according to the CTCAE and had symptoms scored as 4–13 according to the FACT/GOG-Ntx. All six patients had hypoesthesia in a globes and/or socks distribution; four had additional neuropathic breakthrough pain, and three had clinical motor involvement.

As for the start of CNT in relation to the duration of chemotherapy, 4 cases were during chemotherapy, and 2 cases were within half a year after last chemotherapy.

Some improvement of CIPN was found in 2 of the cases after CNT in CTCAE grading, but all of the patients showed improvement in the FACT/GOG-Ntx score. Hypoesthesia in a globe and/or stocking distribution was improved in all six patients. All four patients who complained of breakthrough pain showed apparent improvement (Table 2). Especially 2 of 6 patients with CIPN treated with CNT after they stopped chemotherapy improved after only 1 or 2 times of CNT.

In all of the cases, multiple drugs were used for CIPN. Also, Japanese traditional (Kampo) medicines were prescribed in two cases.

As well as peripheral neuropathy, improvement in some symptoms other cancer treatment-related symptoms was obtained after initiating CNT. Improvement of edema was obtained in 5 cases, fatigue and constipation in 4 cases (67%), and other symptoms in 6 cases.

### 3.2. Case Report

A 66-year-old man (Patient 5) presented with occult blood from the rectum and visited a hospital in May 2007. He was referred to Kanazawa University Hospital and was diagnosed with rectal cancer (RaT2N1M0, stage IIIa). He was initially treated by operation without adjuvant chemotherapy because of the earthquake. Three years after the operation, metastasis was found in S8 and caudal lobe of the liver. He was treated by chemotherapy with SOX + Bev(oxaliplatin, S-1, bevacizumab) regimen for recurrent liver metastases. After 3 courses of chemotherapy, he developed grade 1 CIPN and grade 2 pigmentation of extremities in CTCAE. After 5 courses of SOX + Bev regimen, chemotherapy regimen was changed to FOLFIRI + Bev (folinic acid, fluorouracil, irinotecan, bevacizumab) because of the severe CIPN. Although he stopped oxaliplatin for 12 months, symptoms of CIPN were not improved and were even exacerbated with FOLFIRI + Bev. He decided to stop all chemotherapy, in July 2010, which led to the growth of liver metastasis. Chemotherapy with FOLFIRI + Bev was restarted in November 2011, but symptoms of CIPN were the same. CNT was started in August 2012. After 2 times of CNT, he demonstrated a dramatic improvement in pain, numbness, and discomfort of lower extremities and he could easily walk. After 6 times of treatment, FACT/GOG-Ntx score was improved to 2 from 13. The amelioration of his symptoms enabled chemotherapy to be continued.

## 4. Discussion

CIPN is one of the most serious problems in clinical practice, and it sometimes results in the discontinuation of subsequent treatment [1, 2]. CIPN is well known in taxanes, platinum analogues, vinca alkaloids, and molecular target drugs such as bortezomib [3]. Neuropathy by taxanes stems from damage to microtubules of the neuraxis, mainly developing from gloves-and-socks type sensory disturbance. It is commonly associated with a decrease in reflection, vibratory sensation, muscle ache, and muscle weakness [4]. Platinum analogues such as cisplatin and oxaliplatin damage nerve cells directly, followed by damage to the neuraxis. With oxaliplatin, acute accumulation-related disorder is detected, and acute peripheral neuropathy is induced by a low-temperature stimulus that is known to be reinforced [6].

In this investigation, some improvement of CIPN was found in all of the cases after CNT. CIPN of 2 patients in concurrent chemotherapy was not exacerbated, which may indicate the effectiveness of its prophylactic role. In 4 patients who stopped chemotherapy, CNT was effective on peripheral neuropathy even 2 years after last chemotherapy. CIPN may spontaneously disappear over time in some cases [16] and in some patients in our study as well, but it will be difficult while undergoing the responsible chemotherapy, as well as after more than one year. Patient 5 demonstrated remarkable improvement in pain, numbness, breakout pain, and discomfort of his legs.

Although several prospective clinical trials have indicated acupuncture to be effective in painful diabetic neuropathy (PDN) and human-immunodeficiency-virus- (HIV-) related painful neuropathy [17–20], no known clinical trials have investigated the intriguing potential of acupuncture for the relief of breakout pain in CIPN, which has a dominant position among side effects. Various treatments have been tried, but few of them have demonstrated sufficient effects so far. In the present study, the usefulness of CNT for CIPN by experienced specialist, including the advantage of making adjustments to acupuncture point according to the patients' sho and safety of CNT compared with inserted acupuncture methods, is suggested. Several studies [17–20] have demonstrated that acupuncture is effective in the treatment of CIPN, with fewer adverse effects than analgesic drugs.

From a perspective of Japanese traditional medicine, acupuncture is based on the premise that there are patterns of qi flowing throughout the body, and blockage of qi leads to illness. Qi is defined as a kind of vital energy that is immaterial or invisible in narrow definition and is simultaneously both immaterial and material in wide definition. From the scientific point of view, the proposed putative mechanisms of acupuncture involve regulation of the nervous system, stimulation of the immune system and alteration of brain chemistry causing the release of various neurotransmitters and hormones. Some evidence has shown that acupuncture is a safe pain-control method with minimal side effects, and interest in acupuncture as an adjunctive therapy has grown rapidly in recent years among the general public as well as the scientific and academic communities.

Several human and animal studies have examined the neurochemical basis of acupuncture for pain control, and although no single theory is sufficient to completely explain all the effects of acupuncture, the mechanisms can be partly explained in terms of endogenous pain inhibitory systems. Acupuncture excites receptors or produces rhythmic discharges in nerve fibers activating both the peripheral and central nervous systems, resulting in the release of various neurotransmitters [21–24]. The exact effects of acupuncture depend on point selection and type of stimulation [25].

Serotonergic pathways have been implicated in pain relief and have been useful in relieving discomfort in neuropathy [26]. Thus, it has been hypothesized that acupuncture might work synergistically with serotonergic therapy to relieve neuropathic pain.

According to the research of Litscher et al. [27], acupuncture may increase the blood flow in the extremities. Increased blood flow to the vasa nervorum and dependent capillary beds supplying the neurons may contribute to nerve repair with measurable improvement of axons or myelin sheaths. In addition, the symptomatic effect of acupuncture may reflect morphological changes in the anatomy of peripheral nerves and also complex derangements of central and peripheral regulation [28]. It is not certain whether CNT has the same effect as inserted acupuncture, but those findings in acupuncture give us some suggestion on the mechanism of effect of CNT.

The reason that quite a few randomised controlled trials of acupuncture failed to show the significant effects is partially because of the real effect of no-insertion acupuncture. For example, a randomised controlled trial compared acupuncture and amitriptyline with their respective placebo controls showing that the sham device had greater effects than the placebo pill on self-reported pain and severity of symptoms over the entire course of treatment [29]. In this study, we showed the effect of “placebo” acupuncture, CNT. It might be better to reconsider the method of “placebo” acupuncture in the design of randomised controlled trials.

In this investigation, it is difficult to evaluate everything because of the small study population. We did not evaluate the efficacy after cessation of CNT. It is said that the effect of CNT continues about a week and the accumulative effect is reported in some other researches on acupuncture, so as in this study group. It should be investigated in our next research. However, we think that the potential of CNT for CIPN was clearly indicated. Another shortcoming of this study is the remarkable effect of CNT for pain breakthrough. The benefit of the application of CNT by specialists for CIPN was suggested for the first time in this study.

## 5. Conclusion

There are few standard therapies for CIPN, a condition that often leads to discontinuation of chemotherapy and to deterioration of the subsequent QOL. CNT may improve the symptoms of CIPN and associated side effects during the course of chemotherapy and even after a long interval since the last chemotherapy. CNT might be considered as one of the safe and effective alternative methods for CIPN.

## Figures and Tables

**Table 1:** Patient characteristics.

	Case					
	1	2	3	4	5	6
Age/sex	70/F	54/F	66/M	57/M	66/M	73/F
Performance status	0	0	0	0	0	0
Primary lesion	Colon	Breast	Colon	Colon	Colon	Colon
Chemotherapeutic agents	Oxaliplatin	Docetaxel paclitaxel	Oxaliplatin	Oxaliplatin	Oxaliplatin	Oxaliplatin
Regimen of chemotherapy	XELOX + Bev	DOC PAC	mFOLFOX	mFOLFOX + Bev	SOX + Bev FOLFIRI + Bev	XEROX + Bev
Past operations	Yes	Yes	Yes	Yes	Yes	Yes
Tumor-baring	Yes	No	Yes	Yes	Yes	Yes

XELOX: oxaliplatin, capesitabine.

Bev: bevacizumab.

SOX: oxaliplatin, S-1.

FOLFIRI: folinic acid, fluorouracil, irinotecan.

FOLFOX: folinic acid, acid, fluorouracil, oxaliplatin.

DOC: fluorouracil, epirubicin, cyclophosphamide, docetaxel.

PAC: paclitaxel, cyclophosphamide, doxorubicin.

**Table 2:** Results of symptoms scores pre- and post-CNT and clinical evolutions of patients.

	Case					
	1	2	3	4	5	6
CTCAE						
Before	2	1	2	1	2	1
After	2	1	1	1	2	0
FACT/GOG-NTX						
Before	11	6	10	4	13	9
After	5	2	5	4	2	4
Breakthrough pain						
Before	4	0	2	3	3	0
After	0	0	1	1	1	0
Patients' evaluation	Improved	Improved	Improved	Improved	Improved	Improved
Last responsible chemotherapy (month ago)	Concurrent 12	26	16	Concurrent 16	Concurrent 12	Concurrent 1
Adverse effect of CNT	None	None	None	None	None	None

Breakthrough pain: 0 (None)~4 (Very severe).
